# Mechanical Properties of Crumb Rubber and Basalt Fiber Composite Modified Porous Asphalt Concrete with Steel Slag as Aggregate

**DOI:** 10.3390/polym12112552

**Published:** 2020-10-30

**Authors:** Chao Chai, Yongchun Cheng, Yuwei Zhang, Bing Zhu, Hang Liu

**Affiliations:** College of Transportation, Jilin University, Changchun 130025, China; chaichao18@mails.jlu.edu.cn (C.C.); chengyc@jlu.edu.cn (Y.C.); zhubing18@mails.jlu.edu.cn (B.Z.); liuhang19@mails.jlu.edu.cn (H.L.)

**Keywords:** styrene-butadiene-styrene, porous asphalt concrete, pavement performance, steel slag, SEM analysis, XRF spectroscopy

## Abstract

This paper studies the mechanical properties of porous asphalt concrete with styrene-butadiene-styrene (SBS) polymer modified bitumen as the binder, steel slag as the aggregate and crumb rubber and basalt fiber as modifiers. First, the appearance, mechanics, chemical composition and high-temperature stability of steel slag were studied by some equipment. Then, three kinds of porous asphalt concrete with SBS polymer modified bitumen as binder were produced, Namely, crumb rubber modified porous asphalt concrete (CR-PAC), basalt fiber modified porous asphalt concrete (BF-PAC), and basalt fiber and crumb rubber composite modified asphalt concrete (CM-PAC). Finally, the properties of the three kinds of modified PACs were studied through the Marshall test, freeze-thaw splitting test, low-temperature splitting test, permeability test, and creep test. The results showed that the crush value and abrasion value of steel slag are 15.1% and 13.5%, respectively; it has excellent strength and abrasion. In addition, the steel slag shows a porous structure and it provides an interface basis for a better bond with bitumen. For the three PACs, the results showed that the Marshall stability, water stability, and low-temperature crack resistance of CM-PAC are all the best Furthermore, CM-PAC has better rutting resistance than two single modified PACs, based on creep test results. The CM-PAC in this study can be used as a new type of pavement material.

## 1. Introduction

Porous asphalt concrete (PAC) has been widely studied in recent years because it can better cope with the adverse effects of rainfall on traffic [1,2,3,4]. In addition to greatly reducing the risk of driving in rainy days [5,6,7,8], PAC also has good noise reduction performance [9,10,11,12] and heat absorption performance [13,14,15,16]. Although the permeable pavement paved by PAC has many good service performances, its comprehensive mechanical properties are worse than traditional dense graded asphalt mixtures (such as AC, SMA, etc.) due to its inherent large void structure characteristics [17,18]. Zeng et al. [19] studied the effects of different aggregates and different modifiers on the performance of different asphalt mixtures (AC, SMA, PAC); their results showed that PACs are more prone to stripping. Wu et al. [20] summarized the research results on open-graded friction course (OGFC) in recent years; they pointed out the reasons for the limited application of OGFC in a large range, and proposed future research directions. Xu et al. [21] studied the permeability of different types of asphalt mixtures (AC, SMA, OGFC) under freeze-thaw cycles, and the results show that OGFC exhibits the worst water stability.

Aggregate is an important part of the solid phase of asphalt mixture, and its properties directly affect the performance of the mixture [22,23,24,25]. The natural stones commonly used in asphalt mixture aggregates include basalt, limestone, granite and so on [26,27,28,29]. Natural stones with excellent mechanical properties are good aggregates for preparing asphalt mixtures. However, the over-exploitation of natural stone in some areas has also caused some problems, such as a shortage of natural stone, environmental pollution and so on [30,31,32]. Therefore, researchers began to seek a new type of pavement material with excellent engineering characteristics that can replace natural stones, such as industrial waste steel slag, oil shale, cinder, fly ash. It is worth noting that steel slag has been extensively studied due to its unique formation process and excellent engineering characteristics among alternative materials.

Liu et al. [33] studied the feasibility of using steel slag in the pavement base, as well as the mechanical properties and durability of concrete with different steel slag content. In addition, the microstructure and element distribution of concrete were studied by scanning electron microscope (SEM) and energy dispersive X-ray spectroscopy (EDS). The results showed that concrete with 50% steel slag content had the best performance. Zhu et al. [34] discussed the low-temperature crack resistance of PAC with different steel slag content, and used acoustic emission technology to monitor the splitting process of specimens in real time. It was found that the low-temperature crack resistance of PAC is significantly improved after using 100% steel slag. Hasita et al. [35] studied the fatigue properties and creep properties of asphalt mixtures made of steel slag and the test results revealed that the fatigue life, elastic modulus and rut depth resistance of asphalt mixture made of steel slag are 1.6, 1.4, and 1.4 times higher than those of asphalt mixture made of natural aggregate, respectively. The above studies have shown that the application of steel slag in pavement materials has good engineering performance and has a large application space.

PAC has higher requirements for the viscosity of bitumen. High-viscosity bitumen can make aggregate and bitumen bond better, greatly increasing the mechanical properties of the mixture and the Cantabro particle loss resistance of pavement surface. As an excellent binder, SBS polymer modified bitumen has higher viscosity and better high temperature stability. In addition, the properties of PAC can be further improved by adding modifiers [36,37,38]. Among commonly used modifiers, fibers and rubber powder modifiers have been extensively studied. Wang et al. [39] studied the various properties of basalt fiber modified PACs; the analysis of a series of pavement performance experiments and micro-experiment results showed that the three-dimensional network system formed by basalt fibers in PAC significantly improves the mechanical properties of PAC. Cheng et al. [40] optimized the amount of basalt fiber and rubber powder in PAC by the response surface method, and verified the improvement effect of basalt fiber and crumb rubber on the performance of the mixture through pavement performance experiments; its results paved the way for environmentally friendly road construction. Jiao et al. [41] used acoustic emission technology to study the cracking characteristics of crumb rubber modified PAC. Their work showed that the crumb rubber and styrene–butadiene–styrene modified PAC has a more uniform force and a more stable internal structure; these results were found through the analysis of the results of splitting, compression experiments and acoustic emission parameters. The above studies have shown that fibers and crumb rubber are potentially good modifiers for asphalt mixtures.

In summary, the purpose of this study is to analyze the mechanical properties of a new type of composite-modified PAC. First of all, from the perspective of environmental protection, steel slag was selected as an aggregate, and its physical and chemical properties were explored with modern instruments. Furthermore, the applicability of steel slag as aggregate is also discussed. Then, from the perspective of using modifiers, crumb rubber and basalt fiber were used as modifiers to further study the mechanical properties of SBS-modified PAC. Finally, a comprehensive study of the modified PAC is carried out through pavement performance experiments.

## 2. Materials and Methods

### 2.1. Raw Materials

#### 2.1.1. SBS Polymer Modified Bitumen

The bitumen used in this study is SBS-modified bitumen and the amount of SBS modifier is 4% of the bitumen mass. The technical indicators provided by the manufacturer are shown in Table 1.

#### 2.1.2. Steel Slag

The steel slag used in this experiment is provided by a material company named Shengyuan, Jilin, China. The particle size range of steel slag is from 0.075 to 13.2 mm, which conforms to the particle size distribution of PAC-13. The steel slag was further screened and stored before specimens were made. According to the Test Methods of Aggregate for Highway Engineering (JTG E42-2005) [42], the density of steel slag measured for each grade is shown in Table 2.

#### 2.1.3. Crumb Rubber

The crumb rubber used in this article has a fineness of 40 mesh and is produced by the Hongda Chemical Plant in Jilin, China. The information provided by the manufacturer is shown in Table 3.

#### 2.1.4. Basalt Fiber

The basalt fiber used in this experiment has a length of 6 mm and a diameter of 13 μm, and more information provided by the manufacturer is shown in Table 4.

### 2.2. Tests and Methods

This research mainly focuses on the physical and chemical properties of steel slag and engineering mechanical properties of the modified PACs made of steel slag. The specific experiments are described as follows.

#### 2.2.1. Crush Value Test

The crush value is a measure of the ability of stone to resist crushing under the condition of gradual loading, and it is used to evaluate the applicability of aggregates in pavement engineering. According to the standard (T 0316–2005) [42], first, weigh 3 kg of steel slag of 9.5–13.2 mm after drying and record it as m_0_, put it into the test mold three times, and fully vibrate 25 times each time. After the upper surface of the test mold is flattened, the indenter is placed in the test cylinder, and the test mold is placed on the press, uniformly loaded to 400 kN at a rate of 0.67 kN/s, and unloaded after maintaining for 5 s. Finally, the crushed sample is sieved with a standard sieve of 2.36 mm. Record the quality of fine material passing the 2.36 mm standard sieve as m_1_. The crush value of the steel slag was calculated according to Equation (1), and three sets of parallel experiments were carried out.

(1) $$Q=\frac{m_{1}}{m_{0}}\times 100$$

where m_0_ is the quality of the sample before crush value test, m_1_ is the quality of the fine material passing the 2.36 mm sieve after the test.

#### 2.2.2. Scanning Electron Microscope (SEM) Test

**Figure 1:**
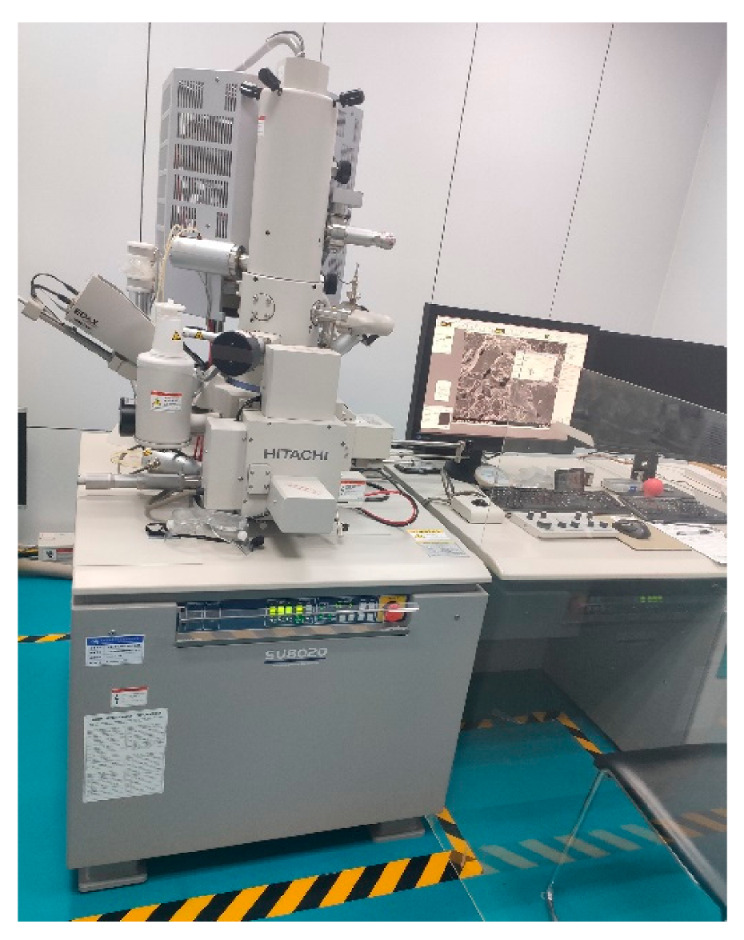
Scanning electron microscope (SEM) test equipment.

Aggregates with rough surfaces are more suitable for PACs because of their greater friction. In order to explore the texture characteristics of the steel slag surface, this paper uses a SEM to conduct a microscopic study of the steel slag. The SEM was produced by Hitachi, Tokyo, Japan. The surface morphology of the crude steel slag was studied through different magnifications. Figure 1 shows the SEM used in the experiment.

#### 2.2.3. X-ray Fluorescence Spectroscopy (XRF) Test

**Figure 2:**
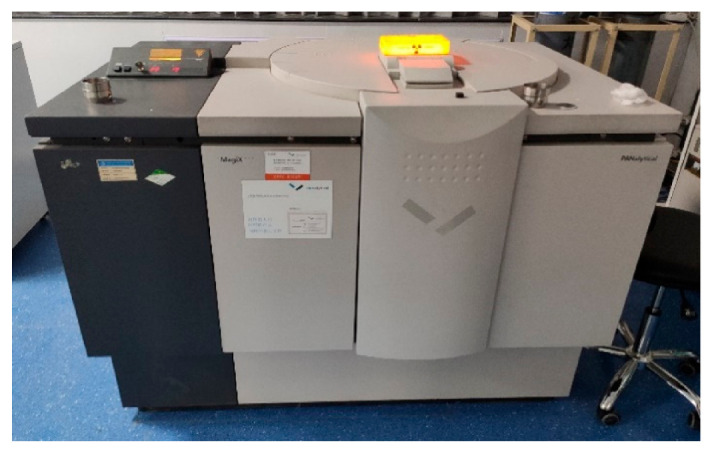
X-ray fluorescence spectroscopy (XRF) test equipment.

XRF was used to further understand the chemical composition of steel slag in this experiment, from the perspective of material composition. XRF is currently the most commonly used instrument for qualitative and quantitative analysis of chemical components. It has the characteristics of fast measurement speed and high sensitivity. The instrument used in the test was produced by PANalytical, Almelo, the Netherlands, and the steel slag sample used was steel slag powder ground from crude steel slag and the powder particle size was less than 80 μm. The chemical composition and proportion of steel slag can be obtained by analyzing the test results. Figure 2 shows the experimental site of XRF.

#### 2.2.4. Simultaneous Thermal Analysis (STA) Test

**Figure 3:**
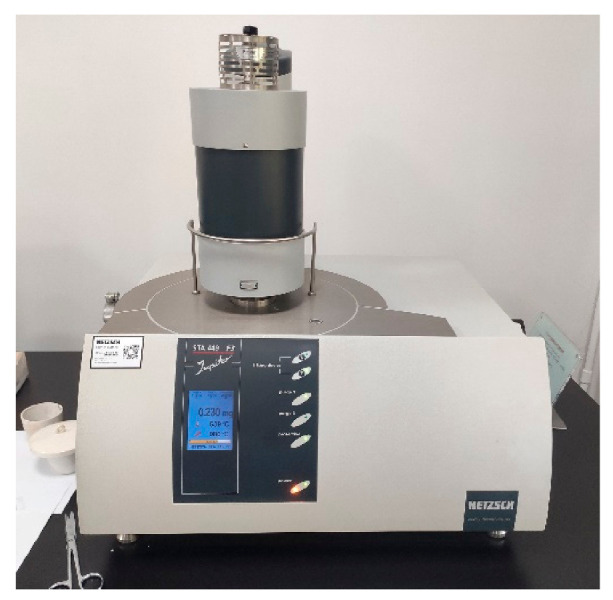
Simultaneous thermal analysis (STA) test equipment.

Sufficient high temperature stability is a necessary property for aggregate, to study the high-temperature performance of steel slag used in this test, this paper studied the steel slag through an STA experiment. The instrument used in the experiment is the STA449F3, produced by NETZSCH in Bavaria, Germany. Its basic principle is that the balance displacement caused by the change of sample weight is converted into electromagnetic quantity. This tiny amount of electricity is amplified by an amplifier and recorded; the amount of electricity is proportional to the change in weight of the sample. Therefore, when the measured substance sublimates, vaporizes, or loses crystal water during the heating process, the quality of the measured substance will change. At this time, the thermogravimetric curve will change. The sample used was steel slag powder, and the experimental temperature rose from room temperature to 900 °C. Figure 3 shows the experimental site of STA.

#### 2.2.5. Design of PAC Gradation

**Figure 4:**
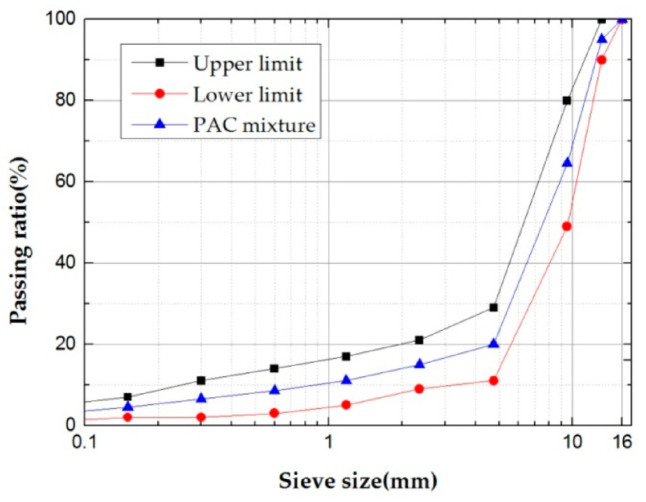
Curves of the porous asphalt concrete (PAC) mixture.

The gradation type of PAC used in this experiment is PAC-13, according to the specification (JTG E20-2011) [43] and the previous research of our group [40], the gradation curve selected in this study is shown in Figure 4. In addition, after determining the steel slag gradation, three different modified PACs were prepared; they are crumb rubber modified PAC (CR-PAC), basalt fiber modified PAC (BF-PAC), and crumb rubber and basalt fiber composite modified PAC (CM-PAC). The amount of crumb rubber in the three modified PACs is 11% of the bitumen mass, and the amount of basalt fiber is 0.45% of the mixture mass. Taking a Marshall sample as an example, the composition quality of the three mixtures are listed in Table 5.

#### 2.2.6. Marshall Test

According to the above mixing ratio, three groups of standard PAC Marshall specimens were prepared, and a Marshall test was performed on the three specimens. The instrument used is a Marshall stability tester with force and displacement sensors, which can record the stability and flow value of each specimen. The specific experimental process refers to the specification (0709–2011) [43].

#### 2.2.7. Freeze-Thaw Splitting Test

The freeze-thaw splitting test was used to evaluate the water stability of the three kinds of PACs, and the specimens used were standard Marshall specimens. The specific experimental process refers to the standard (T0729–2011) [43]. There are three parallel test pieces for each type of PAC. The freeze-thaw splitting tensile strength ratio is calculated according to Equation (2).

(2) $$TSR=\frac{R_{T2}}{R_{T1}}\times 100$$

where *R_T_*_2_ is the splitting tensile strength value after the freeze-thaw cycle, and *R_T_*_1_ is the splitting tensile strength value without the freeze-thaw cycle.

#### 2.2.8. Permeability Test

**Figure 5:**
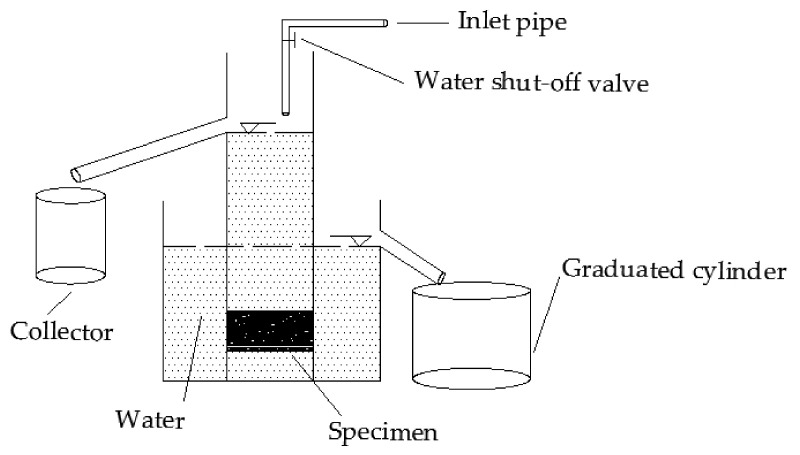
Schematic diagram of the permeability tester apparatus.

A self-made water permeability measuring device was used to study the water permeability of three PACs. Figure 5 shows the schematic diagram of the water permeability tester.

The constant water head method is adopted, and the water head height is 15 cm. After adjusting the water inlet valve to make the head height constant, then record the time required for 5000 mL of water to flow out of the overflow tank. The permeability coefficient can be calculated according to Equation (3).

(3) $$K=\frac{QL}{At\Delta h}$$

where *K* is the permeability coefficient (cm/s); *Q* is the amount of the water permeating through the specimen (5000 mL); *L* is infiltration length (cm); *A* is cross-sectional area of the specimen (cm^2^); *t* is the time from the start of water collection to the filling of 5000 mL of water; and ∆*h* is the water head difference (15 cm).

#### 2.2.9. Low-Temperature Crack Resistance Test

In this study, the low-temperature splitting test was used to evaluate the low-temperature crack resistance of the three modified PACs. The test pieces used were standard Marshall specimens. All test pieces have been kept in a −10 °C environmental box for five hours before the start of the experiment. The test loading rate was 1 mm/minute. According to the standard (0716–2011) [43], the splitting tensile strength, splitting stiffness modulus and splitting tensile strain of each PAC can be calculated.

#### 2.2.10. Creep Test

**Figure 6:**
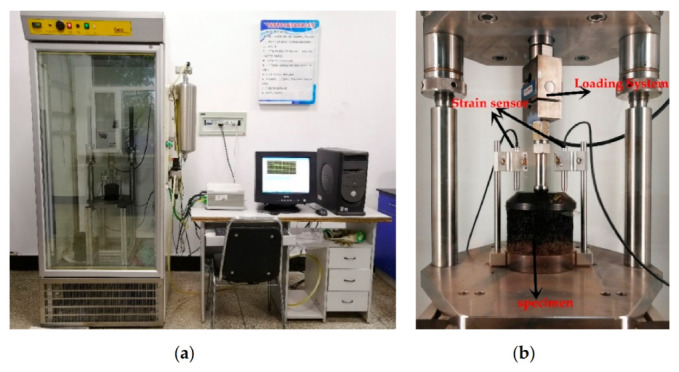
The creep test site; (**a**) General diagram of the test site; (**b**) The test piece in creep test.

The creep test can evaluate the deformation resistance of asphalt mixture under a certain temperature and stable load. In this study, creep experiments were performed on Marshall specimens made of three modified PACs. The experiment temperature was 50 °C, the applied load was 240 kPa, and the experiment time of each specimen was 3000 s. Figure 6 shows the experimental site.

After the creep test is completed, the results are fitted by the classic Burgers model, and further analysis can be directly carried out through the parameters. A typical Burgers model is as in Equation (4).

(4) $$\varepsilon (t)=\sigma_{0}[\frac{1}{E_{1}}+\frac{t}{\eta_{1}}+\frac{1}{E_{2}}(1-e^{\frac{-E_{2}t}{\eta_{2}}})]$$

## 3. Results and Discussion

### 3.1. Analysis of Crush Value and Abrasion Value Results

Table 6 shows the results of the crush value and abrasion value of steel slag. It can be seen from the experimental results that the crush value and abrasion value are 15.1% and 13.5%, respectively; this meet the requirements of the specification [44], which are less than 26% and 28%, respectively. This shows that the steel slag has good strength and wear resistance and can be used as the aggregate of the mixture for further research.

### 3.2. Analysis of SEM Test Results

**Figure 7:**
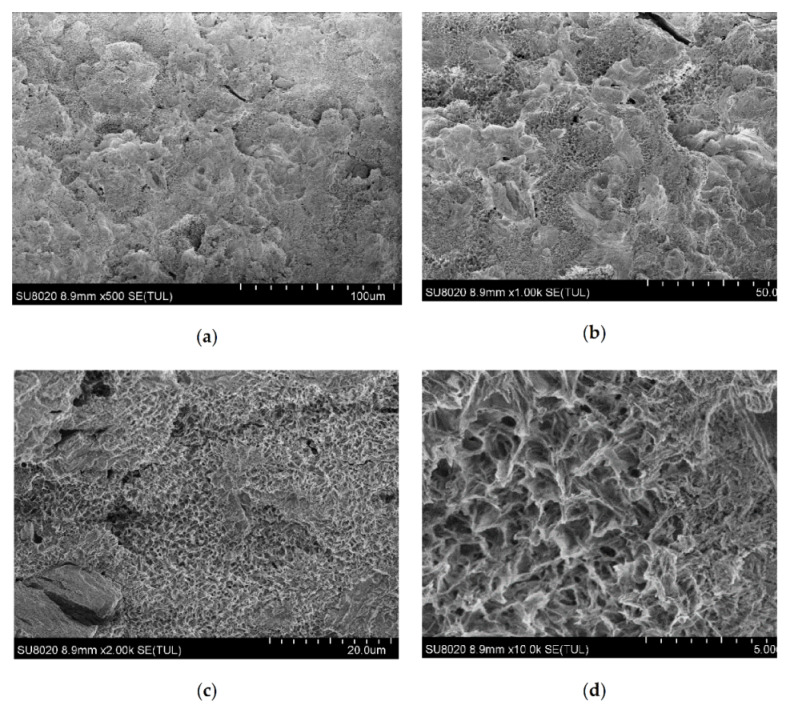
The SEM test results; (**a**) minimum identification length = 100 μm, (**b**) minimum identification length = 50 μm, (**c**) minimum identification length = 20 μm, and (**d**) minimum identification length = 5 μm.

Figure 7 shows the SEM results of different magnifications on the surface of the crude steel slag. It can be seen from Figure 7a,b that when the minimum accurate identification length in the figure is 100 and 50.0 μm, the surface of the steel slag presents a very obvious disordered “peak and valley” shape, which is consistent with the macroscopic observation. Different from the smooth surface of natural aggregate, the surface of steel slag is very rough and uneven.

As shown in Figure 7c,d, the minimum accurate identification lengths in the figure are 20.0 and 5 μm. It can be seen in Figure 7c that the surface of the steel slag presents a fine porous structure, and the radius of the holes is about 10 μm. Figure 7d shows the further enlargement effect of Figure 7c.

It can be seen that the surface of the steel slag is uneven and exhibits obvious porous characteristics through the SEM pictures with different magnifications, which provides the basis for a good bonding effect with bitumen.

### 3.3. XRF Test Result Analysis

Table 7 shows the results of XRF of the steel slag used in this experiment. Among all the elements, the top five proportions from high to low are Ca (53.9%), Fe (21.2%), Mg (9.1%), Si (7.7%), and Al (2.3%). These five elements are mainly metal elements and account for 94.2% of all elements. From the perspective of oxides, the top five oxides are CaO (48.4%), Fe_2_O_3_ (17.7%), SiO_2_ (12.2%), MgO (11.7%), and Al_2_O_3_ (3.3%). These five oxides also accounted for 93.3%. From the perspective of oxide composition, the steel slag has stable chemical properties, and chemical reactions are not prone to occur under normal temperature.

### 3.4. Analysis of SAT Test Results

**Figure 8:**
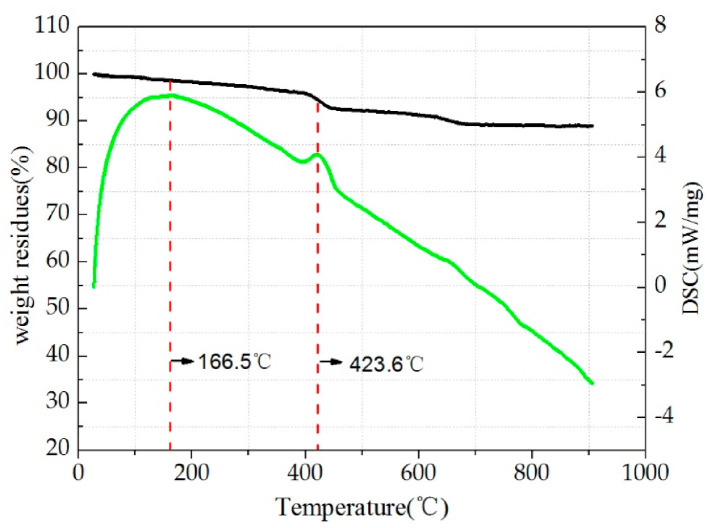
TG-DSC curve of steel slag.

Figure 8 shows the TG-DSC (thermogravimetric analysis-differential scanning calorimetry) curve of steel slag. It can be seen from the figure that when the experimental temperature reaches 166.5 °C, a large exothermic peak appears in the DSC curve, while the corresponding TG curve does not show a significant quality change, which shows that from the room temperature to 166.5 °C, the liquid water inside the steel slag was gradually evaporating, resulting in a slow decline in the quality of the sample. The evaporation continues until the temperature reaches 400 °C. When the experimental temperature reached 423.6 °C, another small exothermic peak appeared in the DSC curve, and the quality of the sample also appeared to undergo a more obvious drop at this time. This may be caused by the decomposition of crystal water inside the sample.

In summary, the chemical properties of steel slag are very stable before 400 °C. Generally, the mixing temperature of asphalt mixture does not exceed 180 °C. Therefore, from the perspective of thermal stability, steel slag is suitable as an aggregate of the asphalt mixture.

### 3.5. Analysis of Marshall Test Result

According to the Marshall test results in Table 8, the air voids of the three types of PAC are all within 20%–22%. The air voids of CM-PAC are the smallest, with a value of 20.4%. The Marshall stability of the three modified PACs all meet the requirements of the specification, and the stability of CM-PAC is largest with a value of 8.94 kN. At the same time, the flow value of CM-PAC is also the largest.

The addition of crumb rubber and basalt fiber reduces the air voids of the CM-PAC. This is because the crumb rubber and the basalt fiber used in this experiment have smaller sizes, which occupy the voids of PAC to a certain extent. In addition, under the combined effect of crumb rubber viscosity increasing and basalt fiber structural bridging, the Marshall stability of CM-PAC has been significantly improved, and the flow value is the same.

### 3.6. Analysis of Freeze-Thaw Splitting Test Result

Table 9 shows the results of the freeze-thaw splitting test; it is found that these three modified PACs are all have values greater than 85%, which shows that all three PACs meet the minimum requirements of the specification. Specifically, the freeze-thaw splitting strength ratios of CR-PAC and BF-PAC are 88% and 89%, respectively. For CM-PAC, the freeze-thaw splitting strength ratio reached 92%, which reveals that under the combined action of crumb rubber and basalt fiber, the combination of bitumen and aggregate in PAC further improves the water stability of the mixture.

### 3.7. Analysis of Low-Temperature Splitting Test Result

**Figure 9:**
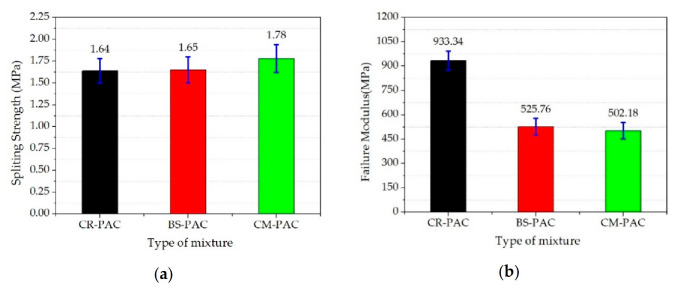

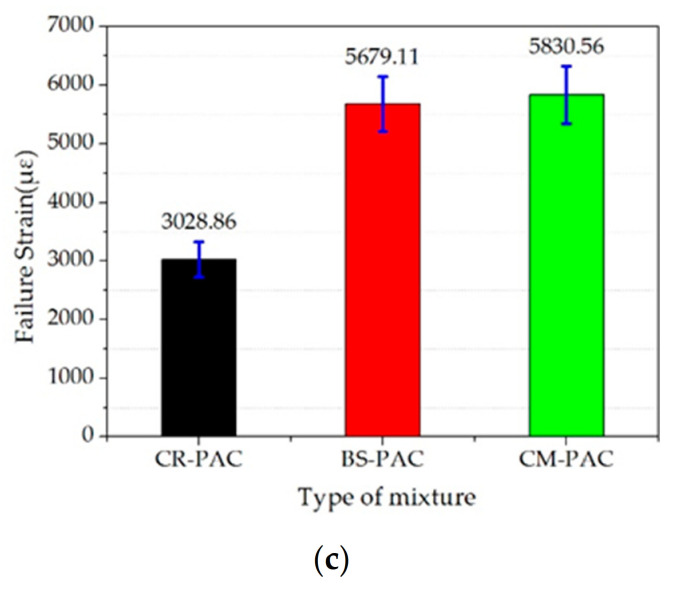
Low-temperature splitting test results of three modified PACs: (**a**) splitting strength; (**b**) failure modulus; (**c**) failure strain.

Figure 9 shows the results of the low-temperature splitting test. Compared with CR-PAC and BF-PAC, the low-temperature splitting strength of CM-PAC is slightly improved. This shows that under the joint action of crumb rubber and basalt fiber, the low-temperature splitting load resistance of CM-PAC has been improved.

In addition, the failure stiffness modulus of BF-PAC and CM-PAC is significantly lower than that of CR-PAC, and their failure strain is obviously increased. This reveals that both of them are easier to deform under the condition of low-temperature splitting, and their ultimate deformation values have been significantly improved. The main reason is that the basalt fiber forms a three-dimensional network structure in PAC, which makes the force of the mixture more uniform. This reinforcement effect of this basalt fiber effectively improves the tensile properties of the mixture.

### 3.8. Analysis of Water Permeability Test Results

Table 10 shows water permeability test results of three PACs. It can be seen that the water permeability coefficient of CM-PAC is 0.287, which is somewhat reduced compared to the values of CR-PAC and BF-PAC, but still meets the requirements of the specification.

This difference in water permeability results occurs because the crumb rubber and basalt fiber are in powder form and fine crumb form, respectively. After being added to PAC, they fill in the voids to a certain extent.

### 3.9. Analysis of Creep Test Results

**Figure 10:**
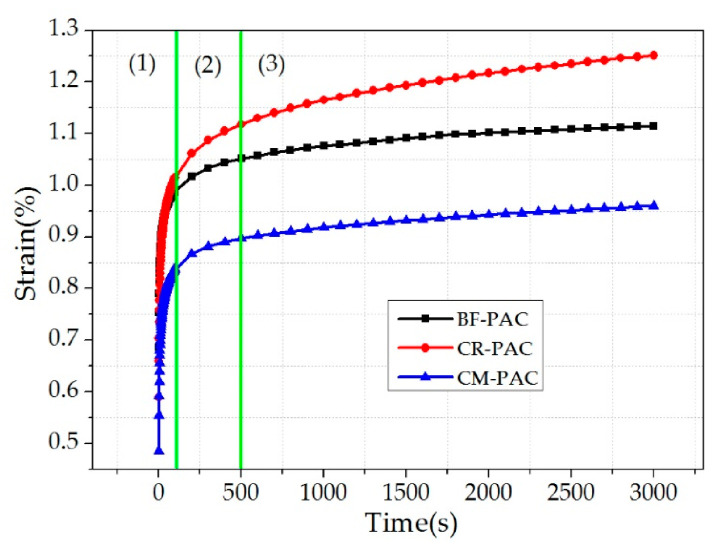
Creep test results of three PACs.

Figure 10 shows the creep test results of three PACs. From the “time–strain” diagram, these three types of PAC can be divided into three stages. The first stage is the rapid growth stage of strain. This stage is approximately from the beginning of the experiment to 120 s. In the second stage, the rate of strain gradually slowed down and this stage is mainly for the experiment to be carried out between 120 and 500 s. The third stage is the stable growth stage of strain; during this stage the deformation rate tends to be constant, and the strain shows a steady increasing trend. This stage continues until the end of the experiment. In addition, it can be seen from Figure 10 that the strain of CM-PAC in all three stages is the smallest, and it can be considered that CM-PAC has the best high-temperature deformation resistance.

In order to quantify the deformation characteristics of the three PACs in the creep stage from the parameters, the creep results were fitted by a four-parameter Burgers model. The fitting results are shown in Table 11.

It can be seen that the fitting coefficients of the three groups of PAC samples are not less than 0.94, which shows that the fitting effect is better. For the parameters E_1_ and η_1_, CM-PAC’s values are 0.43 and 4455.16, respectively, which are the largest among the three groups of PAC samples. This shows that under the same temperature and load conditions, the instantaneous deformation and permanent deformation of CM-PAC are the smallest. For the parameter τ, the value of CM-PAC is 17.72, which is the smallest among the three groups of PAC, which shows that the CM-PAC needs the shortest time during elastic deformation recovery process.

It can be seen that the high-temperature deformation resistance of CM-PAC is the best through the analysis of experimental results and Burgers fitting parameters. The application of crumb rubber can improve the elastic properties of the mixture to a certain extent. In addition, the “bridging” structure formed by basalt fiber in the mixture has a better force–transmission effect. The high- temperature performance of CM-PAC has been significantly improved under the joint modification of crumb rubber and basalt fiber.

## 4. Conclusions

This paper uses a series of modern experimental instruments to study the properties of steel slag and three modified PACs prepared from steel slag as the aggregate and SBS polymer modified bitumen as the binder. Investigations included the physical and chemical properties of steel slag and the pavement performance and viscoelastic properties of the mixture. Through the analysis of the test results, the following conclusions can be drawn:

1. The results of crush value and abrasion value of steel slag meet the requirements of response specifications, indicating that the steel slag used in this experiment has sufficient strength and abrasion resistance. In addition, the steel slag shows a porous structure with unevenness, which provides an interface basis for a better bond with bitumen.
2. The oxide chemical composition of the steel slag used in this experiment mainly includes CaO (48.4%), Fe_2_O_3_ (17.7%), SiO_2_ (12.2%), MgO (11.7%), and Al_2_O_3_ (3.3%). When the temperature is lower than 400 °C, the chemical properties of steel slag are stable and chemical reactions do not easily occur, therefore it can be used as the aggregate of the mixture.
3. According to the road performance results, the Marshall stability, water stability, and low- temperature crack resistance of CM-PAC are all the best; this shows that under the joint action of crumb rubber and basalt fiber, the pavement performance of PAC can be further improved.
4. The air voids of CM-PAC are reduced compared with two single modified PACs, which leads to a certain decrease in water permeability. However, the water permeability coefficient of the CM-PAC still meets the requirements of the specification.
5. Under 50 °C and certain compressive load conditions, the instantaneous deformation and long-term deformation of CM-PAC are the smallest, which shows that CM-PAC has better high-temperature resistance to deformation than two single-modified PACs.

## Figures and Tables

**Table 1:** Technical indicators of SBS-modified bitumen.

Indicators	Results	Specification
Penetration (25 °C, 0.1 mm)	67.3	60–80
Ductility (5 °C, cm)	35.3	≥30
Softening point (°C)	66.7	≥55
Elastic recovery (25 °C, %)	92.1	≥65
Flash point (°C)	267	≥230

**Table 2:** Densities of steel slag.

Properties	13.2 mm	9.5 mm	4.75 mm	Fine Steel Slag
Apparent specific density (g/cm^3^)	3.50	3.56	3.53	3.39
Gross volume relative density (g/cm^3^)	3.34	3.38	3.34	3.33

**Table 3:** Technical indicators of crumb rubber.

Indicators	Results	Specification
Apparent density (g/cm^3^)	1.16	1.1–1.3
Metal content (%)	0.038	<0.05
Moisture content (%)	0.40	<1
Fiber content (%)	0.38	<1
Ash content (%)	5.1	≤8

**Table 4:** Physical properties of basalt fiber.

Index	Diameter	Length	Specific Gravity	Tensile Strength
Units	μm	mm	g/cm^3^	MPa
Value	13	6	2.63	2320

**Table 5:** The composition quality of the three mixtures (one Marshall sample).

Mixtures	Mineral (g)	SBS Modified Bitumen (g)	Crumb Rubber (g)	Basalt Fiber (g)
CR-PAC	1225.49	49.02	5.50	0.00
BF-PAC	1223.27	51.38	0.00	5.35
CM-PAC	1213.77	54.74	6.14	5.35

**Table 6:** Crush value and abrasion value of steel slag.

Indicators	Results	Specification
Crushed stone value (%)	15.1	≤26
Los Angeles abrasion (%)	13.5	≤28

**Table 7:** XRF test results.

**Element**	**Na**	**Mg**	**Al**	**Si**	**P**	**S**	**Cl**	**K**	**Ca**
Percentage	0.239	9.095	2.313	7.671	1.087	0.339	0.109	0.082	52.987
**Element**	**Ti**	**V**	**Cr**	**Mn**	**Fe**	**Zn**	**Sr**	**Nb**	**Ba**
Percentage	0.518	0.26	0.261	3.552	21.249	0.015	0.044	0.009	0.17
**Oxide**	**Na_2_O**	**MgO**	**Al_2_O_3_**	**SiO_2_**	**P_2_O_5_**	**SO_3_**	**K_2_O**	**CaO**	**TiO_2_**
Percentage	0.253	11.694	3.296	12.219	1.811	0.607	0.068	48.448	0.516
**Oxide**	**V_2_O_5_**	**Cr_2_O_3_**	**MnO**	**Fe_2_O_3_**	**ZnO**	**SrO**	**Nb_2_O_5_**	**BaO**	
Percentage	0.277	0.227	2.686	17.668	0.011	0.028	0.007	0.107	

**Table 8:** Marshall test results.

Mixtures	Air Voids (%)	Marshall Stability (kN)	Flow Value (mm)
CR-PAC	21.3	8.18	2.25
BF-PAC	21.1	8.29	2.96
CM-PAC	20.4	8.94	3.34
Specification	≥18	≥5.0	2−4

**Table 9:** The freeze-thaw splitting test results.

Mixtures	Splitting Strength (MPa)	Splitting Strength (after Freeze-Thaw) (MPa)	Freeze-Thaw Splitting Strength Ratio (%)
CR-PAC	0.61	0.54	88
BF-PAC	0.63	0.56	89
CM-PAC	0.65	0.59	92
Specification	-	-	≥85

**Table 10:** The results of the permeability test.

Types of Mixture	Pervious Time(s)	Specimen Height (mm)	Voids in PAMs (%)	Permeability Coefficient
CR-PAC	86.0	63.5	21.3	0.308
BF-PAC	88.0	63.9	21.1	0.302
CM-PAC	92.0	63.8	20.4	0.287
Chinese standard	-	-	≥18	≥0.28

**Table 11:** Results fitted using the Burgers model.

Mixtures	E_1_	η_1_	E_2_	η_2_	τ	R^2^
CR-PAC	0.34	2605.48	0.75	21.03	28.04	0.96
BF-PAC	0.31	4396.81	1.13	24.01	21.25	0.94
CM-PAM	0.43	4455.16	0.88	15.59	17.72	0.96
